# Beyond Psychiatric Illness: Paediatric Autoimmune Neuropsychiatric Syndrome Presenting as Psychosis

**DOI:** 10.1192/bjo.2025.10720

**Published:** 2025-06-20

**Authors:** Akshaya Dixit, Saikat Roy

**Affiliations:** 1 Central and Northwest London NHS Foundation Trust, London, United Kingdom; 2 West London NHS Trust, London, United Kingdom

## Abstract

**Aims:** Paediatric Autoimmune Neuropsychiatry Syndrome is a rare condition with exact prevalence not known due to dearth of large-scale population studies. Its diagnostic as well as treatment guidelines are not yet validated. There are a few international guidelines but none available in the UK yet.

**Methods:** The referral of a 15-year-girl presenting with symptoms suggestive of PANS to our team prompted us to write this case report. The patient was brought in to the A&E by her parent after an episode of bizarre and chaotic behaviour at school, which included her throwing belongings at the staff while being emotionally labile. On assessment she reported symptoms suggestive of paranoid delusions, delusional perception, auditory and gustatory hallucinations, thought broadcasting and delusional memory. She was also taking more frequent showers, with clothes on. Her sleep was disrupted.

Extensive blood and radiological investigations initially didn’t yield any positive findings. Antipsychotic (olanzapine) was initiated and the patient’s mental state quickly improved. Two days later blood investigations showed raised antistreptolysin O titres (200–400 U/Ml). Hence, antibiotic (phenoxymethypenicillin) was initiated. The patient was discharged and followed by our team in the community.

The patient continued to present with residual psychotic symptoms which fluctuated in the community setting. But the overall functioning significantly improved.

**Results:** The acute onset of psychosis, characterized by behavioural disturbances, anxiety, sleep disruption, irritability, and emotional lability, along with elevated antistreptolysin O titres, suggested a diagnostic formulation aligning with PANS.

**Conclusion:** The case underscores the importance of considering PANS as a differential diagnosis in school-aged children presenting with symptoms of OCD, first episode of psychoses or unexplained emotional lability.

